# Innovative High-Pressure Fabrication Processes for Porous Biomaterials—A Review

**DOI:** 10.3390/bioengineering8110170

**Published:** 2021-11-01

**Authors:** Mythili Prakasam, Jean-François Silvain, Alain Largeteau

**Affiliations:** CNRS, Univ. Bordeaux, Bordeaux INP, ICMCB, UMR 5026, F-33600 Pessac, France; jean-francois.silvain@icmcb.cnrs.fr (J.-F.S.); alain.largeteau@u-bordeaux.fr (A.L.)

**Keywords:** bioceramics, metallic implants, biodegradable polymers, porous biomaterials, high pressure processing, freeze isostatic pressure

## Abstract

Biomaterials and their clinical application have become well known in recent years and progress in their manufacturing processes are essential steps in their technological advancement. Great advances have been made in the field of biomaterials, including ceramics, glasses, polymers, composites, glass-ceramics and metal alloys. Dense and porous ceramics have been widely used for various biomedical applications. Current applications of bioceramics include bone grafts, spinal fusion, bone repairs, bone fillers, maxillofacial reconstruction, etc. One of the common impediments in the bioceramics and metallic porous implants for biomedical applications are their lack of mechanical strength. High-pressure processing can be a viable solution in obtaining porous biomaterials. Many properties such as mechanical properties, non-toxicity, surface modification, degradation rate, biocompatibility, corrosion rate and scaffold design are taken into consideration. The current review focuses on different manufacturing processes used for bioceramics, polymers and metals and their alloys in porous forms. Recent advances in the manufacturing technologies of porous ceramics by freeze isostatic pressure and hydrothermal processing are discussed in detail. Pressure as a parameter can be helpful in obtaining porous forms for biomaterials with increased mechanical strength.

## 1. Introduction

Porous materials [1,2,3,4] have special properties in comparison with their dense counterparts. Porous materials have potential applications as high-temperature filters, thermal gas separators, lightweight structural components, biomaterials and as thermal structural materials. Other applications, where porous materials with specific chemical compositions and tailored microstructures are required, include electrodes and supports for batteries and solid oxide fuel cells, scaffolds for bone replacement and tissue engineering, heating elements, chemical sensors, solar radiation conversion, among others. Fabrication methodology plays a vital role in obtaining the pore structure, morphology and density of pores in the porous body and their essential properties such as mechanical properties are intricately dependent on each other. Various processing methods [5] are available for fabricating the porous structures such as replication of polymer foams by ceramic dip coating, foaming of aqueous ceramic powder suspensions, pyrolysis of preceramic precursors, partial sintering by pressure-less sintering, and sintering of ceramic powder compacts with pore-forming sacrificial phases. Frequently, the fabrication methodologies involve employing high-temperature techniques. Employing high temperature can be detrimental, especially in the case of biomaterials, as most of them consist of active therapeutic molecules or water molecules in their structure. To increase the structural stability, temperature is used very often for the porous ceramics. Pressure is another important thermodynamic parameter that can help in fabricating porous biomaterials obtained at low temperature with increased mechanical strength. The pressure parameter can be successful in the case of decontamination of biomaterials that are thermosensitive. The advances in the processing techniques helps in obtaining the porous body with homogeneous pore distribution and desired pore morphology and by loading with active biomolecule or for drug-delivery systems. Cell proliferation in the porous materials is dependent mainly on the composition of the material, pore size distribution and morphology and open interconnected pores in the order of 50–150 µm. The structural stability of the porous biomaterials is, in general, brittle, which is intricately dependent on parameters such as porosity volume fraction, pore size and pore structure. Innovative high-pressure processes can be a viable solution to consolidate and to increase mechanical strength. Porous biomaterials have attracted great interest as scaffolds for tissue engineering, particularly bioactive ceramics and glasses, as they are able to bond to the host tissues. From the mechanical aspect, the degree of porosity is vital than pore size and scaffolds with porosities greater than 40% can be used as trabecular bones. For in vitro and in vivo performances, pore size is more important and co-existence of macropores and micropores helps in good vascularization of the porous biomaterial. Porosity is an important consideration for nutrient transfer, and promotes cell migration and proliferation, leading to regeneration and integration.

Unique properties arise while employing the pressure parameter for processing as the free volume of the system decreases with increasing pressure. Temperature effects act with an increased kinetic energy as well as increased free volume. According to the Le Chatelier-Braun principle [6], pressure affects primarily the volume of a system, while temperature changes cause volume as well as energy changes. High-pressure processes act as an important tool for improving the investigations on chemical bonding and consequently leads to induced physico-chemical properties. High pressure can lead to structural transformations and help the synthesis of novel materials. In both cases, the condensation effect (ΔV < 0 between precursors and the final product) is the general rule. In addition, through the improvement of the reactivity, high pressures can lead to materials that are not reachable through other chemical routes. Very well-known high-pressure processing is high-pressure torsion (HPT) which refers to the processing of metals whereby samples are subjected to a compressive force and concurrent torsional straining process leading to grain refinement, often at the nanometer level, with high mechanical strength. Several processes can lead to a reduction of the pore volume under pressure such as particle fragmentation and rearrangement, deformation of the zones of contact between particles until closed porosity is formed, and shrinkage of individual pores. When an external pressure is applied to packed powder particles, force is exerted on the particle contacts leading to localized particle deformation. The deformation of the particle contacts under the action of the effective pressure causes instantaneous plastic yielding of the contact zone or stress directed diffusion process from the contact area to the pore surface. At relatively low pressure, the diffusion mechanisms tend to contribute more to densification than power-law creep which, in turn, predominates at high hot isostatic pressure (HIP). This is because the driving force for the diffusion processes is much less sensitive to the effective pressure than the rate of dislocation creep. The application of pressure (as a “driving force”) manifests itself in different ways such as reduced sintering temperature leading to conservation of grain size and sintering the high-pressure structural phases. This review focuses on employing pressure as processing parameter to obtain porous biomaterials. Innovative high-pressure processing for porous biomaterials is presented and the current state of the art for metals, polymers and ceramics is discussed.

## 2. Discussion on Different Biomaterials

This section discusses various class of materials that are used as porous biomaterials.

### 2.1. Biodegradable Polymers for Tissue Engineering

Strategies in tissue engineering involve scaffolds, differentiated and undifferentiated cells and growth factors. Tissue engineering requires scaffolds that are biocompatible, biodegradable, non-toxic and in addition should provide appropriate mechanical support and adequate surface properties (required for adhesion, proliferation and differentiation of cells). Among the different options present for scaffolds, polymers are preferred for their ability to degrade by the enzymes present in the body, minimize inflammatory reactions and non-toxicity. These polymers are classified such as natural polymers and synthetic polymers [7].

Natural polymers are extracted from tissues like collagen and plants. Synthetic polymers, on the other hand, can be obtained from the polymerization of the monomers. 3D porous polymer scaffolds play a vital role in the tissue engineering and regenerative medicine. Different types of synthetic polymer, such as polylactic acid (PLA) and its copolymers, polylactide-co-glycolide (PLGA) and PLGA-polyethylene glycol (PEG), offer processing flexibility and less immunological issues in comparison to the extracellular matrix (ECM). Various synthetic polymers for biomaterials are discussed in the following section.

#### 2.1.1. Synthetic Polymers for Biomedical Applications

Polyglycolic acid (PGA)—PGA is one of the biocompatible and biodegradable aliphatic polyesters that is used for medical applications. Kobayashi et al. [8] reported on the PGA-collagen nanocomposite which was vascularized within 5 days after implantation in animal models. Patrascu et al. [9] reported on PGA-hyaluronan (HA) composites on their chondrogenic potential of implants containing the mesenchymal stem cells (MSCs) in vitro and in a rabbit articular cartilage defect model. From the literature review it can be found that PGA composites are suitable as scaffolds for cartilage regeneration and blood vessels.

Polylactic acid—PLA is a thermoplastic aliphatic polyester, biodegradable and bioabsorbable with two optical isomers such as L- and D-lactic acid. PLA is widely used in orthopedic devices, mesh, screws, pins and rods as implants. Lin et al. [10] reported on the bone regeneration on hydroxyapatite (HAp) and chitosan coated with PLA. Mi et al. [11] reported on the possibility of using polyurethane (PU) and PLA at different ratios as the scaffold for the tissue engineering. The PU-PLA at different ratio offered the possibility to obtain surface roughness, mechanical properties and biocompatibility. PU-PLA possibility to tune the characteristics apt for soft and hard tissue regeneration.

Polycaprolactone (PCL)—PCL is a biocompatible, bioresorbable and biodegradable polyester. PCL is used in dental splints, targeted drug delivery and medical implants in addition to tissue engineering. Zheng et al. [12] reported on the characteristics of PCL adapted for the use of cartilage and in other tissue regenerations. Other composites of polyvinyl alcohol (PVA), HAp and PCL nanofibers reported by Uma Maheswari et al. [13] show the potential of PCL as a biocompatible scaffold for bone and cartilage regeneration.

Poly (lactic-co-glycolic acid)—PLGA is a biocompatible, biodegradable copolymer that has potential applications in therapeutic tools, tissue engineering and drug-delivery systems. Junmin Qian et al. [14] studied the influence of PLGA on enhancing the mechanical strength of the scaffolds in the PLGA-nano HAp biocomposite due to the modification of the crystallinity of the PLGA polymer in the composite. They played a vital role in initiating osteoblasts essential for bone regeneration.

Poly (*N*-isopropylacrylamide) (PNIPAM)—PNIPAM is a thermosensitive polymer with unique physical and chemical applications in tissue engineering for regenerating damaged bone tissues and in drug delivery. Sa-Lima et al. [15] studied the capacity of poly (*N*-isopropylacrylamide)-g-methyl cellulose (PNIPAM-g-MC) as thermo reversible hydrogel as a 3D scaffold for cartilage regeneration. PNIPAM, when forming composites with other compounds, can act as a suitable scaffold for tissue engineering applications.

Poly (DL-lactic acid-co-glycolic acid)-g-ethylene glycol (PLGA-g-PEG)—this is a biodegradable and bioresorbable polymer that is employed for tissue engineering and in drug-delivery systems. Sidney et al. [16] reported on the possibility of using PLGA/PEG scaffolds as localized drug-delivery system for bone regeneration.

Poly (caprolactone/ethylene glycol) (PCL-PEG) copolymer—this is a biodegradable and biocompatible polymer that has potential applications in tissue engineering. Niu et al. [17] reported on the possibility of creating a suitable environment for the regeneration of damaged tissue with the high surface area porosity for cell adhesion and cell differentiation. Based on the animal model chosen, it was inferred that PCL-PEG nerve-guide scaffolds had the potential for good peripheral nerve regeneration.

Poly(caprolactone/lactide) copolymer (PCL-PLA)—this is a biodegradable, bioresorbable and biocompatible polymer with various applications in tissue engineering. PCL-PLA copolymer nanofibers were reported to help in regeneration of the damaged tissue and drug-delivery systems. Karimi et al. [18] reported on the electrospun PCL-PLA nanofibers containing thymol helping in wound healing.

#### 2.1.2. Natural Polymers for Biomedical Applications

Polymers originating from biological systems such as plants, animals and microorganisms are classified as natural polymers. Natural polymers are employed for various uses: drug delivery, cosmetics, medical scaffolds and adhesive bandage. Natural polymers are preferred due to their similarity to the host tissue, metabolic compatibility, nontoxicity and low inflammatory reactions. On the downside, natural polymers have high temperature sensitivity and are destroyed prior their melting point. Furthermore, there is high probability to transmit the diseases to human from the natural plant and animal sources. Currently, two types of natural polymer are employed such as polysaccharide-based and protein-based. Chitin, chitosan and alginate are well known polysaccharide based natural polymers. Collagen and gelatin are well-known protein-based natural polymers [19,20,21] widely used. A detailed review on the usage of biodegradable polymers was reported by M. Prakasam et al. [22].

### 2.2. Porous Bioceramics

Bioceramic [23,24,25] with porous morphology is interesting owing its possibility to allow bone tissue growth that leads to fixation of the bioceramic in the implantation site. Porous bioceramics should have biocompatibility, biodegradability, osteoconductivity and good mechanical strength. On the other hand, their applications are limited to non-load bearing bones, fillings and as coating for metallic implants due to their brittle nature, low ductility and poor degradation rate. Β-TCP, which has higher degradation rate in the body than HAp, is combined with different polymers to make a composite that has low fracture toughness. Coating of polymer on the porous bioceramics is one of the methods to improve porous bioceramics mechanical properties. Miyazaki et al. [26] and Miao et al. [27] reported on the improvement of compressive strength of porous bioceramics with silk protein on α-TCP and PLGA on HA/TCP composite, respectively. Microporosity and nanoporosity present in the bioceramics has a strong influence on their biological response such as protein adsorption, cell adhesion and permeability of the biomaterial to the physiological fluids. Bioceramics with osteogenic and antimicrobial properties by incorporation of copper, zinc and silver was investigated [28]. The possibility of incorporating copper, strontium, zinc, cobalt, boron and silicon improved the osteogenesis property. Porous bioceramics of CaPs fabricated at low temperature offers the possibility to incorporate active therapeutic molecules. By changing the textural property of this porous bioceramic, it is possible to controlled drug-delivery systems or other bone morphogenetic proteins and growth factors. The morphology of the pore and their shape had an influence on the cell behavior for cartilage regeneration. Spherical pores with low permeability had enhanced matrix production and gene expression in vitro compared to cube-shaped pores [29].

### 2.3. Bioactive Glasses

Bioactive glasses (BG) are considered as attractive materials for biomedical applications. Materials consisting of calcium, phosphorous and silicate are classified as BG. The first BG developed was 45S5 [30]; this material is widely used for bone graft applications. BGs are also known for their ability to facilitate osteoblast proliferation and differentiation for bone regeneration. Other types of BG are glass ceramics (S53P4) and borate-based glasses (19-93B3). The biocompatibility of these BGs is dependent on the quantity of silicate component (45–52%) and for initiating the bone grafting process. Interfacial bonding, formed either by degradation or dissolution of activate osteogenesis, is strong in BGs. Borate bioactive glasses are known to degrade faster than silicate bioactive glass; 45S5 BG is osteoconductive and also osteoinductive. BGs with added polymers are used for bone tissue regeneration such as soft-hard tissue interfaces which have complex tissue structure defects. Gelatin-BG scaffolds shows vascularization of the cells in the scaffold pores demonstrating their ability to support cell growth [31]. BG-chitin and BG-chitosan nanocomposites porous scaffolds, prepared by lyophilization with pores in the range of 150–300 µm, demonstrated in vitro behavior with osteoblast like cells showing the adhesion of cells to the pore walls [32]. Other BG-polymer composites, such as PDLLA, P(3HB), PLGA, and PCL-gelatin, showed the possibility of using these BGs as bone regenerative material with good osteoblast adhesion. BG scaffolds are fabricated by various methods such as the one pot synthesis method, melt quenching, sol-gel synthesis, cetyltrimethylammonium bromide (CTAB), polyurethane sponge template method and 3D printing. Porous BGs are fabricated also by sintering. This requires high temperature and 45S5 has a small interval between the glass transition temperature, and the crystallization temperature makes the glass stability region narrow leading to a decrease of mechanical strength of the scaffold.

### 2.4. Metallic Biomaterials

Very well-known metallic [33] biomaterials are stainless steel, magnesium, titanium, and tantalum which are widely employed for various biomedical applications. Iron based alloys such as Fe–Mn, Fe–P are investigated as biodegradable materials for applications in stents and as bones. Fe alloys provide the required mechanical property and corrosion rate. SS 316 L is a well-known alloy that is used for joint replacement, bone plates and screws. Co–Cr alloys are biocompatible and have high corrosion and wear resistance. These alloys are used in manufacturing of surgical implants, stents and in dental and bone implants. Co–Cr–Mo [34] alloys and carbide dispersed Co alloys are used in hip joints. Ti alloys with aluminum and vanadium were previously widely used. Currently, Ti alloys [35] with tantalum, tin, niobium and zirconium are used based on their non-cytotoxicity, good corrosion resistance and biocompatibility. Ni–Ti alloys with their shape memory properties are used for applications in orthodontic wires, dental bridges, self-expanding stents and in prostheses. Porous Ta alloys [36] are bioactive and are used as coatings and for non-load bearing orthopedic applications. Alloys of Mg [37] with Zn, Zr, Zr-Ru, Pt and Pt alloys, gold and gold alloys both in porous and dense form are used for tissue-engineering applications.

### 2.5. Porous Scaffold Fabrication Methods

Complex architectures of porous scaffolds for tissue engineering are a field that warrants more investigation for innovative fabrication methods. Scaffolds employed in tissue engineering have high porosity and are biodegradable, non-toxic and should aid in cell differentiation. Different methodologies are commonly employed in scaffold fabrication such as solvent casting, freeze drying, gas foaming and electrospinning. Recently, with the advances in manufacturing technology, innovative high-pressure processing called freeze isostatic pressure [38] has been used for fabricating scaffolds for a high porous body with increased mechanical strength. The aforementioned scaffold manufacturing technologies [39,40,41,42,43] are discussed in detail here.

#### 2.5.1. Solvent Casting

Solvent casting involves casting the solute from the solution by dipping the mold into it and giving it enough time to dry/evaporate to form the solute layer. This method is disadvantageous due to the use of toxic solvent which denatures the protein. Here, the polymer is dissolved in the solvent containing uniformly distributed salt particles of specific size and the solvent is evaporated leaving behind the matrix with uniformly distributed salt particles. The salt particles are then leached out to obtain uniform pores. To avoid the influence of solvent on the polymer, the samples are processed and dried in vacuum conditions to eliminate the solvent. The samples thus obtained have porous structure.

#### 2.5.2. Freeze Drying

This technique involves the production of porous scaffolds by a sublimation process. The solute and solvent are mixed according to the required concentration and then frozen. The ice crystals formed during the freezing process creates the porosity, which are then subjected to lyophilization under high vacuum. The pore size and the shape can be altered based on the pH and freezing rate. The samples thus yielded have controlled porosity and 3D pore structure but lack high mechanical strength. On the other hand, this process does not involve utilization of high temperature and controlled solidification in the single direction to create uniform homogeneous pore structure.

#### 2.5.3. Gas Foaming

This technique employs high-pressure CO_2_ gas to create the porosity in the scaffolds. The porosity and its structure depend on the amount of gas used. CO_2_ gas at high pressure saturates the polymer with gas, causing the dissolved CO_2_ to be unstable and it separates from the polymer forming pore nucleation. These pores then decrease the polymeric density by expansion of polymeric volume. This technique does not involve usage of organic components or a requirement of high temperature.

#### 2.5.4. Electrospinning

Currently, electrospinning is a widely used technique for producing continuous fibers in submicron to nanometer scale range. Nanoparticles mixed with polymers are electrospun to produce scaffolds. This technique involves the assembly of nanoparticles through the alignment of fibers and reduce the Gibbs free energy. No functionalization process is required and it is dependent on high electrostatic forces. Various factors such as solution viscosity and flowrate, electric field intensity, work distance and air humidity play a role in the fabrication of scaffolds.

#### 2.5.5. Three-Dimensional Printing

Three-dimensional (3D) printing is currently most used for fabricating porous ceramics with the possibility to customize the design and size of the pores. Surgical tools, custom-made prostheses, dental porcelain, and porous ceramic filters are a few examples of the possibilities of the products made by 3D printing. Three-dimensional printing technology for porous ceramics gives increased flexibility and rapidity as a low-cost sustainable product fabrication alternative. Generally, there are various hindrances in fabricating the porous ceramics such as pore network interconnectivity, poor reproducibility, thin structures and time-consuming processes with the conventional fabrication techniques. Three-dimensional printing offers the possibility to obtain custom-made porous ceramics with computer-assisted design thus providing provisions to make complex porous structures. The porous structure formation by 3D printing can be precisely altered with control of microstructure and optimization of parameters.

#### 2.5.6. Other Processing Techniques

Various other processing routes [44,45,46] such as replica, sacrificial template and direct foaming methods are available for the production of macroporous scaffolds. The polymer replica technique can give open porous structures with pore sizes in the range of 200 µm to 3 mm and the percentage of porosity varies between 40% to 95%. The downside of this technique, however, is the weak mechanical strength occurring during the pyrolysis of the polymer. The wood structure replica technique is the well-known ancient technology used for obtaining porous scaffolds with highly oriented open pores in the range of 10–300 µm with porosities in the range of 25–95%. The manufacturing cost and presence of open pores on the cell walls and high anisotropy are the drawbacks of this technique. Sacrificial templating is also another technique used in the fabrication of macroporous samples. This method involves the removal of the sacrificial template through pyrolysis, evaporation or by sublimation. The slow removal of the sacrificial phase, on the other hand, increases the sample processing time.

### 2.6. Pressure-Assisted Porous Scaffolds Fabrication

This section discusses the various pressure-assisted porous fabrication techniques. However, techniques such as electric current assisted sintering, hot pressing, microwave sintering and pressure-less sintering are not discussed in this review, as they are extensively reviewed elsewhere [47]. Pressure can act as a driving force for diffusion, enhance plastic deformation and improve particle rearrangement for consolidation of materials. Enhancement of hardness and mechanical strength were achieved by severe plastic deformation through grain size refinement. High-pressure torsion (HPT) [48] is one of the well-known techniques to induce large strains under high hydrostatic pressure. This technique is mostly used for consolidation of ceramic, metallic and amorphous materials or even composites [49]. The cold welding process is achieved through application of isostatic pressure on metallic powders arising from their ductility leading to densification by plastic deformation. In the case of compounds such as ZrO_2_ and Al_2_O_3_, high pressure does not induce any plastic deformation due to the repacking of grains under applied pressure. Recently cold sintering has been used for consolidating materials either in porous or dense form [50]. The various mechanism perceived for the cold sintering is attributed to particle dissolution and reprecipitation, plastic deformation and hydrothermal-type process [51,52,53,54,55,56]. Various high pressure processing techniques used for materials processing are given in Table 1 [6,53,57,58,59,60,61,62,63,64,65,66,67,68,69,70,71,72,73,74,75,76], mainly at elevated temperature for various level of pressure regarding the technologies used.

The porous structure can be obtained by applying pressure for short time duration and/or lower temperature compensated by higher pressure (acting as driving Force) in these HP processes after the initiation of the necks, to avoid densification.

#### 2.6.1. Isostatic Pressure at Negative Temperature: Freeze Isostatic Pressure

Cold isostatic pressure (CIP) performed on hydrated CaCO_3_ leads to consolidation of powders at room temperature [66]. In presence of water, this consolidation was presumed to occur either by a dissolution/precipitation process or by plastic deformation. The dissolution is favoured between the grain contacts, where the stress concentration is high with the liquid interface layer while external force is applied to the grain. The precipitation of the dissolute particles resurfaces in the low stress zone. This process is similar to what happens in hydrothermal sintering process. As for metallic powders, in the presence of solvent, plastic deformation occurs. The presence of liquid is necessary for consolidating the materials under high pressure and low temperature. The presence of pressure increases the effect of surface energy to cause diffusion to occur. A similar phenomenon can be considered for snow deposited on the glacier surface which then transforms into firm ice. D.S. Wilkinson [77] studied pressure sintering theory on ice. Based on the study of ice, at low applied pressure, lattice diffusion dominates the temperature but as the pressure is increased, or under yield stress, plastic flow occurs rapidly.

**Figure 1:**
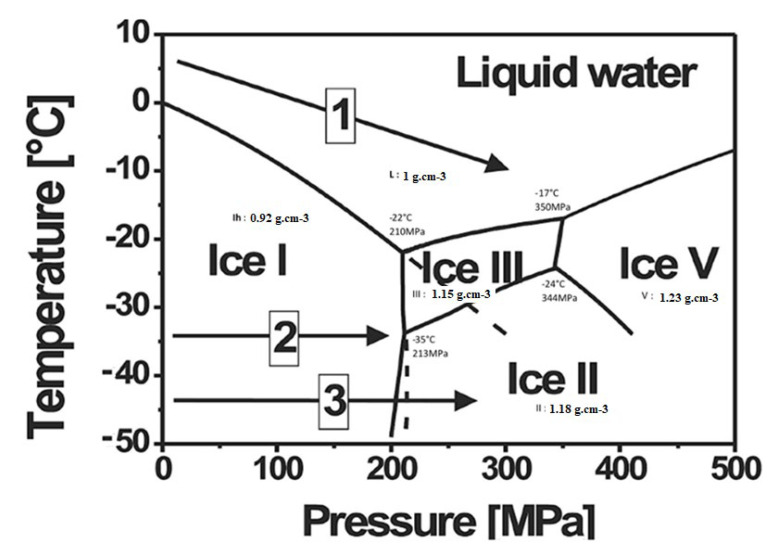
P-T diagram of water and their corresponding densities [78].

Formation of a neck is due to the presence of pressure between the ice crystals; diffusion and plastic flow is evident in nature from phenomena observed in glaciers. To understand ice crystal behavior under pressure, it is essential to know about the phase diagram of water (Figure 1). In 1912, Bridgman [78] reported five different crystalline forms of ice comprising from type I–IV and VI. Among these, type I ice exists in solid form at atmospheric pressure and has low density compared to other types of ice crystal. With the increase in pressure, type I ice shows a decrease in melting temperature up to −22 °C at 200 MPa. When water is transformed into type I ice, their volume increase. In accordance to Le Chatelier’s principle, the increase in pressure causes a decrease in temperature as pressure opposes the increase in volume from the formation of type I ice.

Above 200 MPa, the slope of melting temperature curve is positive. Above 600 MPa, ice forms exist above 0 °C hence freezing is possible at ambient temperature. It is known that compressibility of water is around 8% at 200 MPa and 14% at 400 MPa. By controlling the temperature, it is possible to change the morphology and the size of the ice crystals as observed in pressure assisted freezing and thawing widely applied in food sciences. At low temperatures, activation energy for power law creep is larger, in comparison to lattice diffusion, and grain growth is slow. When porosity concentrations are low, the driving force decreases and the lattice diffusion from grain boundaries to the pores is dominant. Figure 1 shows various pathways possible for the formation of ice crystals along with their densities. Crystallization and melting not only depends on the pressure level and temperature range but also on pressurization and depressurization rates. Control of temperature and the heating/cooling rate is complicated in an autoclave. Recently, in the case of freeze isostatic pressure (FIP), Largeteau et al. [63] have developed an innovative technique, which could be defined as a CIP process at minus temperature. This innovative technique called freeze isostatic pressure (FIP) consists of the application of pressure at minus temperature on a mixture made of powder and pure water (solvent by using mineralizer). Water is used as a template under solid state as ice which is removed at ambient pressure and temperature. In the FIP process, the ice crystals nucleation/growing of water can be controlled. Water used as porogen is removed by sublimation. Prakasam et al. [63] worked on using ice as a template as a porogen (ecological, and safely eliminated from sample) and apply the pressure simultaneously to consolidate the materials. By selecting the appropriate P and T, it is possible to preserve the integrity of the biomaterial containing water through formation of ice crystals. Additional parameters such as rate of compression, decompression, freezing and thawing, determine pore size formed by the ice crystals. The dissolution localized at grains contact favored by a high stress contact point acts like the dissolution and precipitation processes hypothesized in a hydrothermal sintering (HyS) process. The transportation of species along the grain boundary, in the lesser stress contact point, leads to a precipitation on the grain surface and initiates the neck formation in the free spaces between grains, acting like an osmotic pressure effect. Blackford et al. [79] explained this phenomenon in the sintering of ice crystals by transportation. In our case, FIP could be explained by the process where inside the meltwater pressure created at the sliding interface between ice crystals and SiO_2_ particles. The frictional heating generated by the external force on the mixture (powder SiO_2_ + water), was followed by the dissolution of SiO_2_ particles (even if it is low for the chosen temperature) inside this meltwater layer surrounding the ice crystals in contact with particles of SiO_2_. Moreover, SiO_2_ precipitation take place where the constraint is lower by the diffusion between the particles of the meltwater which presents higher mobility than the ice crystals under pressure, and the meltwater deposits SiO_2_ dissolute. Finally, the meltwater freezes inside the free space where the frictional heating does not exist because it is low stressed. The formation of ice templating could be formed inside the mixture by deep-freezing inside a deep-freezer at T < −50 °C rapidly before applying the FIP process for consolidation. The deep-freezing leads to the formation a homogenous and finely crystallized ice inside the mixture.

**Figure 2:**
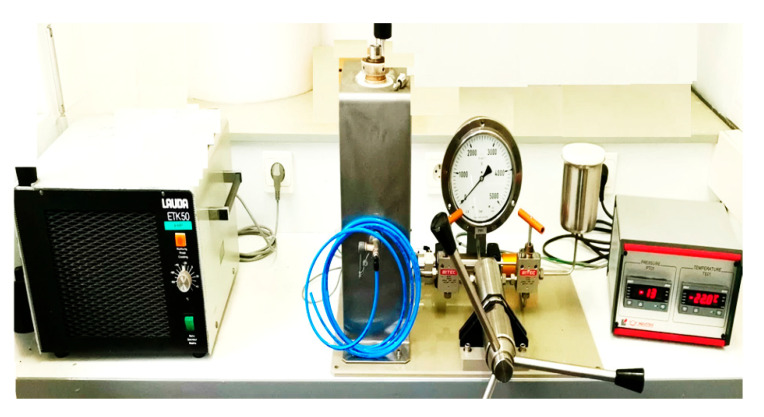
Freeze isostatic pressure equipment at ICMCB.

In summary, we can assume that a hydrothermal reaction is “any heterogenous chemical reaction in the presence of a solvent (whether aqueous or non-aqueous) under liquid state at pressure greater than 1 atm in a closed system”. As the solvent is under liquid phase, even negative temperature could enhance dissolution and precipitation by pressure at the contact point of the grains. This consolidation between grains could be also enhanced by adding binder such as collagen, gelatin, polymer (ex: PVA), and so on. Figure 2 shows the FIP ICMCB equipment.

#### 2.6.2. Isostatic Pressure at Positive Temperature

A pressure exerted by a static liquid or gas propagate is equal in all directions. The material to be compacted is brought into a flexible tube and introduced into a liquid or gas medium. On this medium, a certain pressure is applied so that this pressure acts equally in all directions of the areas of the form. Thus, the material is multidirectional compacted and the compaction of the resulting material is based on the compressibility of the material. Isostatic pressing techniques [80] are classified based on the tool design, temperature and pressure transfer medium used. Cold isostatic pressing (CIP), which is undertaken at ambient temperature, is generally used as a compacting step and requires a subsequent process of sintering to hold the particles together. After compaction by CIP, the sample is called as “Green body”. In hot isostatic pressing (HIP), the material undergoes compacting and sintering, simultaneously. HIP yields higher green bodies densities than other conventional die-pressing techniques. The green body obtained is more uniform and the stresses are not created in the body. Some tensile or compressive stresses may appear depending on the movement of the mold displacement that may occur during non-isostatic compaction. Gas isostatic hot pressing without a mold is used to obtain materials with lesser porosity. Under high gas pressure the open porosity is prevented from closing. Capsule free/mold free hot isostatic pressure yields an open porous body. HIP can be applied after all conventional sintering methods in order to improve the mechanical strength and to reduce the pore size. Under high gas pressure, the surface diffusion is enhanced. Under high pressure, the densification is delayed due to the decrease in the driving force of sintering by enhanced surface diffusion compared to other conventional sintering. Due to well grown necks, HIPed porous materials have high mechanical strength, narrow pore size distribution and high fluid permeability. At high pressure, gas leads to higher density than by gas at a low pressure or under vacuum. HIP enhances the neck formation of the pores and the necks enlarge with little densification leading to an increase in their mechanical strength with narrow pore size distribution and high fluid permeability. HIP porous materials have potential application as biomaterials, filters, grinding wheels, porous detectors for electrochemical analysis.

#### 2.6.3. Gas-Reinforced (GASAR) Technique

This technique is the abbreviation of “gas-reinforced” (Gas + Armirovat (reinforce—in Russian)). This technique [81] involves unidirectional solidification of gas supersaturated melt through eutectic point. Due to their higher gas solubility in the liquid phase, solidification of the metal and nucleation of gas pores occurs simultaneously leading to the formation of an ordered gas-eutectic composition. This phase transformation is very similar to the conventional eutectic reaction. Gas-reinforced metal matrix composites is also known as lotus type or ordered porosity materials, as no evident gas eutectic formation is evidenced. The major advantages of this technique are its improved strength, control of pore shape and orientation, flexibility to yield ordered regular structures, gas and liquid permeability, wide range of pore diameter (from 10 µm up to 10 mm) and ease of fabrication at low cost. Based on the nature of the metal, the formation of pores during solidification is higher due to higher gas solubility and shrinkage phenomenon than in the solid phase. Gas supersaturation in the melt is a prerequisite for the formation of the pores in GASAR. A mixture of active gases if hydrogen or nitrogen and neutral gases such as argon or helium is used in GASAR to have flexibility for formation of porous structure. Usage of neutral gases, due to their insolubility in metal melts, affects the pressure in the gas pores thus help to control the pore size and porosity concentration inside the body. Various metal and alloys were used in this technique to fabricate porous metals, alloys and intermetallics (Al, Be, Cr, Cu, Fe, Mg, Mn, Mo, Ni, Ti, Cu-Al, Al-Si, Ni-Al, Ni_3_Al, TiNi, steels and Fe). The porosity formed in this technique is located at the solid–liquid interface. The possibility of porosity range in this technique depends on the gas diffusion coefficient and gas solubility in the melt. The pore direction is perpendicular to the moving solidification front and the porosity is strongly dependent on partial gas pressures during melt saturation and solidification and the pore diameter is sensitive to solidification velocity.

#### 2.6.4. Hydrothermal Sintering (HyS)

In 1972, the conventional hot pressing in the presence of water in the hydrothermal conditions was defined as hydrothermal sintering. The objective of this technique is to densify and consolidate, in a confined pressure medium (such as autoclave), at lower temperature and in the presence of pressure and solvent. This process offers the flexibility to consolidate dense, porous and layered monoliths. Dissolution and reprecipitation in the presence of solvent and pressure is the process that governs consolidation rather than the solid diffusion process observed in other conventional techniques. The hydrothermal process (if not aqueous solvent: solvothermal) is a deviation of chemical principle of hydrothermal crystal growth (HyCG) used for synthesis of single crystals of alpha-quartz SiO_2_. Hydrothermal conditions favored a chemical reaction at low temperatures due to the effect of subcritical or supercritical state of the hydrothermal fluid. Pressure is exerted from a combination of (1) external uniaxial force applied by a hydraulic press which is localized and high at grain contacts and (2) autogeneous hydrostatic pressure caused by the expansion of the solvent in the autoclave which is isostatically surrounding each grain, following Kennedy’s abacus. The gradient of pressure between grain contacts and inter grains partially or full of water (or solvent in the case of presence of mineralizer) in undercritical (UCF) or supercritical (SCF) state depends on pressure and temperature (P&T) employed during consolidation. The transportation of the species, from the high-stress contact point to the less-stress contact point, will initiate the neck formation through precipitation. This process is based on parameters such as pressure, temperature, state of fluid to judge dissolution and the solvent/mineralizer used. Duration of the hydrothermal reaction also plays the main role in the consolidation process of HyS. By varying the pressure and the temperature, it is possible to sinter the desired phase such as for example amorphous-amorphous or amorphous-crystallized phase. Thus, this innovative HyS process [82] offers the possibility to obtain ceramics of alpha-quartz SiO_2_ with amorphous precursors of silica at lower temperature than phase transition temperature of alpha-beta quartz SiO_2_ which occurs at 573 °C and ambient pressure. Other examples of materials consolidation by HyS are hydrated composition (ex: HAp), porous or dense microstructure, and low chemical reactive compounds such as SiO_2_ and TiO_2_.

#### 2.6.5. Thermosensitive Materials Processing with High Hydrostatic Pressure

The current state of the research field on biocomposites are synthetic materials which are used for repair, replace and/or create an interface between the biological environment. Recent advances in fabrication processes allow the possibility of incorporating active materials such as drug-delivery systems. To date, various radiation sources such as gamma, ultraviolet (UV), e-beam, ion beam was widely used for biocomposites to decontaminate. Usage of polymers as packaging substances is very well known and the decontamination of these biocomposites is undertaken conventionally by irradiation technique. However, the radiation can react with the crosslinking properties of polymers resulting in radical changes of their various functional properties. Another potential alternative innovative technique that can be employed is high hydrostatic pressure (HHP) processing, which is discussed in the present work. In the early 1990s, HHP processing was mainly developed for food to diminish microbiological decontamination to improve shelf-life for consumption. At present, HHP is an emerging technology with widespread applications in Biosciences, including pharmaceutical products, cosmetics and medicine. Non-thermal decontamination, in particular cold decontamination, using pressure treatment, permit the inherent property of the material (food, drug, cream, biomaterial, etc.) to be preserved. The main applications of HHP technology are decontamination at temperature below the usual temperature applied in pasteurization (called also appertization) and at the same time it aids preservation of the inherent properties such as vitamins, color, taste, and smell, texture in foods and on raw materials.

The HHP process consist of a closed high-pressure vessel filled with fluid acting as a transmitting media compressed by pump or by diminishing the volume of the vessel, to apply isostatic pressure. HHP are beneficial aspects includes preserving the therapeutic efficiency of drug in pharmaceutical products and for decontaminating biomaterials in particular for polymers which are thermosensitive. Other promising applications of HHP are that it is foreseen is to decontaminate and/or to impregnate some porous biocomposite with inorganic structure such as thermosensitive polymers (collagen, hydrogel) for decontamination; in other words, decontaminating thermosensitive biocomposites packed in flexible polymer film to avoid direct contact with pressure transmitting media. With the assistance of HHP the packaging surrounding the material to be decontaminated, transmits the pressure homogenously. With such potential applications, it is of high commercial and clinical importance to evaluate HHP processing as a decontamination, up to a sterilization, method at temperature compatible with the stability of sensitive components required for better functional activity. Properties of biocomposite constituted by thermo-responsive hydrogel with a nano/micro encapsulated complex-drug delivery system mean that HHP processing can be a potential new method of decontamination for medical and biocomposites materials. These materials which are sensitive to the currently existing decontamination processes such as gamma irradiation or high temperature methods for a wide range of biocomposites.

### 2.7. Examples of Porous Silica and Porous Copper by Innovative High-Pressure Processing

Silica-based bioactive glasses are used successfully for different bone defects and soft tissue engineering. Silica-based glasses are widely preferred as a biomaterial, but due to the lack of mechanical properties their clinical applications are limited. Silica-based glass is touted to be a third-generation biomaterial for bone tissue regeneration. Silica-based mesoporous materials can be an excellent candidate for controlled drug delivery systems and grafting material for bone regeneration. Macroporous SiO_2_ bioactive scaffolds are required for osteoblast proliferation. Jones and Hench developed 3D bioactive macroporous scaffolds which have poor fracture toughness and pore strength. In the present work we have reported on porous SiO_2_ scaffolds by freeze isostatic pressure (FIP) and this process allows the scaffold and incorporate therapeutic drug molecules to be decontaminated at the same time during fabrication process. Our innovative equipment allows a porous structure of biocomposites (inorganic structure with thermosensible polymer or therapeutic molecules as an example) to be obtained with as a second effect a decontamination (called as sanitization) at low temperature, called cold decontamination (also called Pascalization) by the HPP process.

#### 2.7.1. Freeze Isostatic Pressure (FIP) Processing of Amorphous SiO_2_

**Figure 3:**
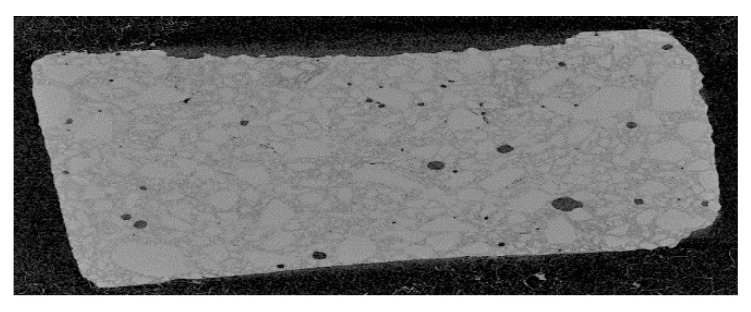
Tomography (internal diameter cut view, diameter: 10 mm) of the sample consolidated by freeze isostatic pressure (FIP) showing a non-uniform distribution of pores and inhomogeneous repartition of solvent (presence of agglomerates).

**Figure 4:**
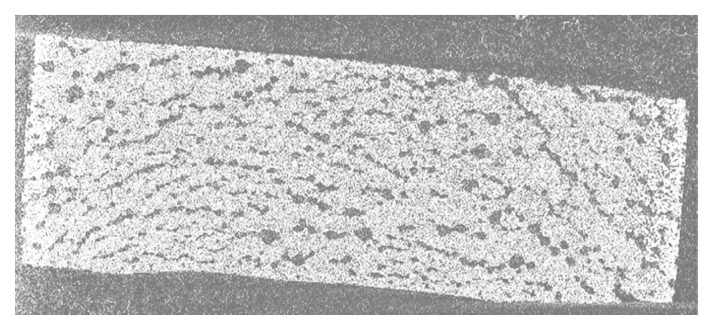
Tomography (internal diameter cut view, diameter = 10 mm) of the sample consolidated by FIP showing an uniform distribution of pores by increasing the content of solvent.

The FIP experiment was undertaken on AlfaAesar amorphous spherical SiO_2_ powder (ref: L16987) with 1.5 µm diameter to manufacture monoliths of porous silica such as biomaterial model which could contain pharmaceutical molecules, mainly destroyed at temperature beyond human corporal temperature. We mixed 0.5 g of AE-SiO_2_ powder with 190 µL NaOH-2M at 1500 bar for 3 h. The choice of the amount of powder and the solvent is important for the study of the material. An excess of solvent will make the pellet brittle and more powder will increase the thickness of the pellet; too thick a pellet is of no interest in testing which is also limited by the volume of the mold. To form a pellet, graphite mold containing the mixture of SiO_2_ + NaOH-2M was placed at −80 °C in the deep-freezer for minimum 1 h (Figure 3). The frozen mixture was then unmolded and sealed quickly inside packaging. The FIP experiment was carried out under the following conditions: the sealed pellet was re-placed in the deep-freezer at −80 °C for 1h, and the pellet was immersed in the pressure transmitter fluid at −40 °C inside the vessel of FIP. The pressure of 1500 bar was applied immediately and held for 3 h. This pellet presents 45% porosity (Figure 4).

**Figure 5:**
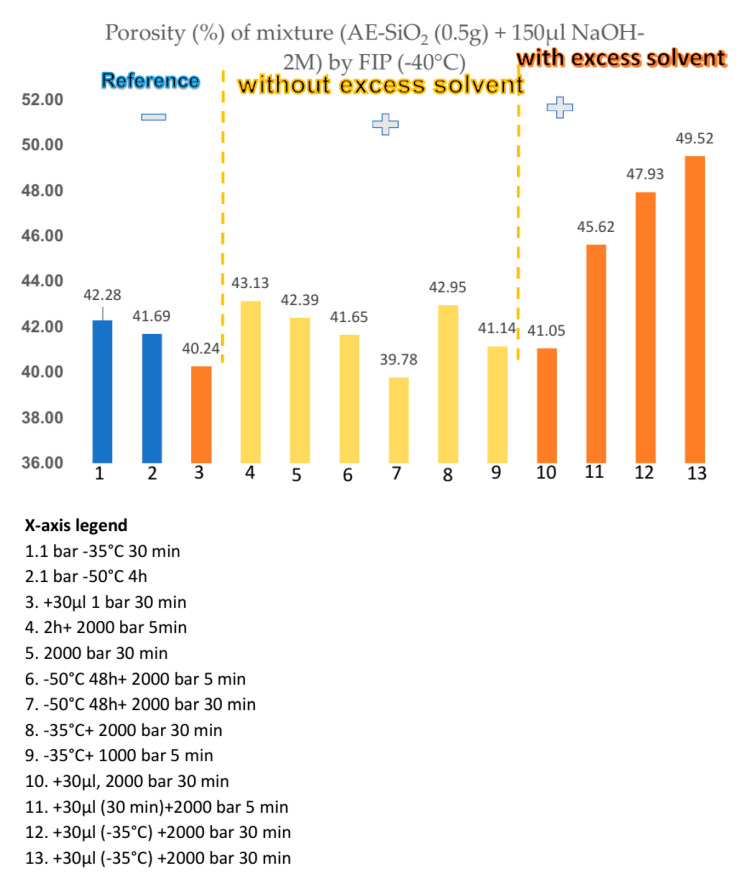
Porosity as a function of various parameter of FIP process.

Tomography analysis allowed us to visualize how the distribution of the solvent is not homogeneous in the powder because there are agglomerates. The increase of solvent quantity led to an increase of porosity, as illustrated in Figure 4. An improvement in mechanical strength was achieved by evaluation: like powder (not reported), impossible to handle (friable, not reported), possible to handle but fragile (−), not breaking by falling 1 m (+). We observed that pressure was increased from 1500 bar to 2300 bar (Figure 5). The pressure, therefore, does not influence the porosity and the strength of the pellet, but it has a beneficial effect on the mechanical strength.

FIP experiments show that porosity is related to the volume of solvent used for preparing the solution (Figure 5), and with the increase of solvent the porosity increases. Porosity was greatly influenced by the freezing time for making the ice crystals. With the increase of pressure the FIP process yielded a good mechanical property.

#### 2.7.2. Hydrothermal Sintering of Porous Amorphous SiO_2_

This HyS process will help for the improvement of consolidation phenomena in comparison with FIP process with the help of temperature above 20 °C. The temperature will increase the dissolution, but the precipitation has to be driven to select the amorphous form, by avoiding the crystallized form which is not biocompatible. The example given below concerns the use of PLA particles as a template to form porous structure after its elimination inside the mixture of AE-SiO_2_ + PLA; PLA is a biodegradable polymer. The final composite AE-SiO_2_ + PLA could be also used like biocomposite.

We used 0.5 g of amorphous SiO_2_ from Alfa Aesar (AE-SiO_2_) for fabricating porous samples with mechanical property after evaluation by: not breaking by falling 1 m (+), and resistant to manual fracture (++).

**Figure 6:**
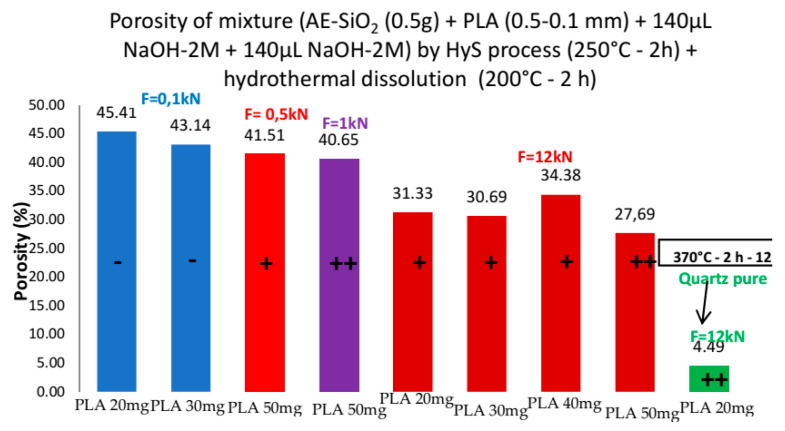
Porosity of AE SiO_2_ by hydrothermal dissolution.

The porosity study was carried out by adding to the mixture (0.5 g AE-SiO_2_ + 140 µL NaOH-2M) different amounts of particles of PLA sieved at 100–500 µm. This biodegradable polymer melts at 150 °C for easy disposal. The study was done at 250 °C to avoid crystallization of the amorphous SiO_2_, which leads in a denser material if the PLA is not added. A second treatment was at 200 °C for 2 h in hydrothermal fluid by using conventional HP vessel with pure water, to eliminate the PLA still present in the pellet by dissolution (Figure 6).

**Figure 7:**
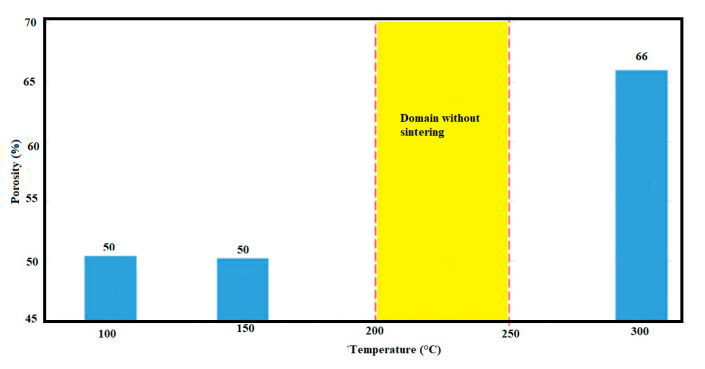
Porosity as a function of temperature in hydrothermal sintering.

The Figure 7 shows that the porosity is low if the force is high because high pressure induces high compactness. The pellet with the lowest porosity value (4.49%) was synthesized at 370 °C for 2 h at 12 kN (1500 bar), leading to complete crystallization, as this is the stability domain of the quartz phase. This high temperature induces a greater dissolution of the PLA. The pellets synthesized in the field of stability of the amorphous phase at low forces (0.1 kN (12 bar) and 0.5 kN (100 bar)) exhibit high porosity but low mechanical strength (friable). On the other hand, the pellet synthesized at 1 kN (127 bar) has both high porosity (40.7%) and very good mechanical strength (not brittle). The content of PLA affects also the mechanical behavior but is compensated by the force applied.

#### 2.7.3. Hydrothermal Sintering of Porous Spherical Copper

Copper was used in the form of particles inside ceramics for antibacterial action and improvement of synthetic bone graft substitutes [4]. Then, copper was studied as a metallic biomaterial model based on the well-defined particles.

The copper powder used had spherical copper grains with a diameter of the order of 50 to 100 μm. Spherical copper is very difficult to compact because the spherical shape does not help with intergranular cohesion. The mechanical strength after uniaxial pressing with 1 g of spherical copper powder was very poor with compression forces varying between 0.4 t and 3 t for a period of 5 min at room temperature. Above 3 t, the pellets may stay in place but collapse after removal of pressure. Water was added as a binder for spherical copper particles, but they still could not stay in place. Copper and water form a heterogeneous mixture (poor wetting of the beads) hence we chose to use PVA (polyvinyl alcohol) with a mass concentration of 50 g/L (powder dissolved in water) as a binder. The samples thus obtained had good mechanical resistance after uniaxial compaction. Furthermore, the pellets were compacted by cold isostatic pressure to increase the mechanical strength. With the increase of force, compactness increases. Porosity of 35% was obtained at 1.2 t and temperature of 350 °C with 7 µL PVA for a dwell time of 2 h.

**Figure 8:**
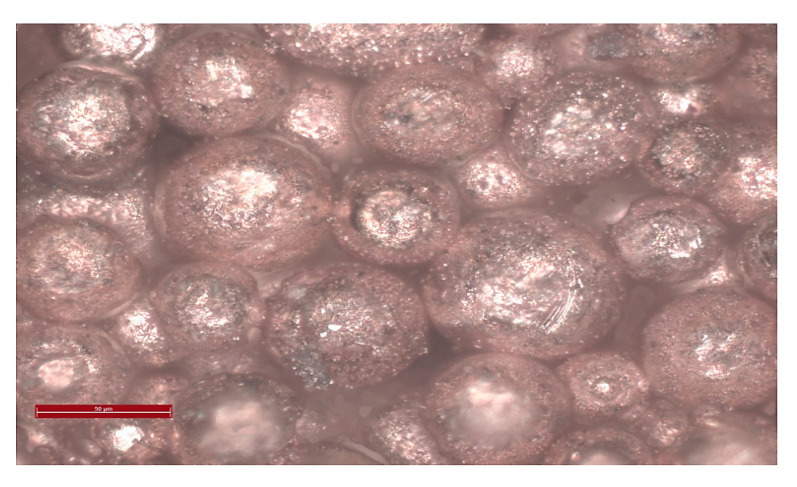
Microstructure of porous Cu samples obtained by hydrothermal sintering.

**Figure 9:**
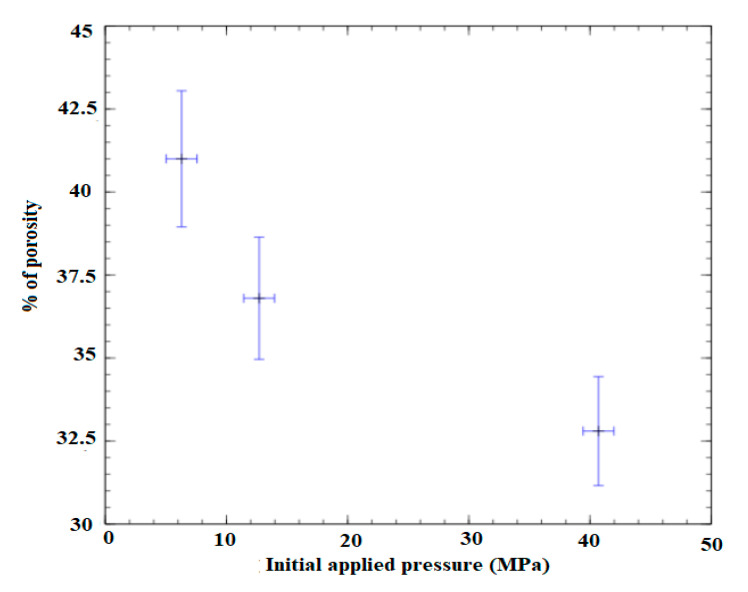
Variation of porosity with respect to initial applied pressure in Cu porous samples by hydrothermal sintering.

The hydrothermal sintering process consists of applying an initial pressure of between 6 and 40 MPa with a rise in temperature to 370 °C, maintained for 2 h on the Cu + H_2_O mixture (Figure 8). A porosity of 40% was obtained. The variation of porosity was observed with respect to the initial pressure applied (Figure 9).

These results obtained show the advantage of high pressure in obtaining porous samples by innovative high-pressure processing. The sections described in 2.6 and 2.7 can be employed with any kinds of biomaterial ranging from metals, polymers, composites and to therapeutic compounds. As high-pressure techniques at low temperatures (FIP, CIP and HyS) can be used for sterilization of the fabricated samples, these techniques at high pressure and low temperature can be employed for clinical applications.

## 3. Summary and Outlook

Porous scaffolds are indicated for tissue engineering in restoring bone defects. The porous scaffolds help cells to attach, proliferate and differentiate to form a desirable new tissue. Various parameters such as composition, structural features such as porosity, pore size and morphology play a vital role in obtaining tissue engineering. The pores and their morphology are essential in judging their mechanical and biological performances. The degree of porosity influences their performances in vitro and in vivo. Several processing techniques are available for fabricating porous biomaterials in the form of polymers, composites, metals and ceramics. Very well-known techniques are gel casting, slip casting, freeze casting and foam/replica methods, electrospinning and in addition to conventional sintering techniques and 3D printing. Pressure and temperature are two thermodynamic parameters essential in engineering the microstructure with controlled porosity and morphology. Various pressure-based innovative techniques are used for fabricating porous scaffolds. Amongst the well-known methods are GASAR, hot isostatic pressure and hot pressing. In recent years, hydrothermal sintering and freeze isostatic pressure have offered possibilities to obtain porous scaffolds of polymers, metals, composites and ceramics at low temperature and high pressure. These innovative techniques can be employed on materials that are susceptible to phase changes at high temperature and for thermosensitive components. FIP allows the possibility to incorporate active therapeutic molecules in the porous scaffolds at low temperature and simultaneously disinfect the scaffold to be directly used for in vitro and in vivo applications. The hydrothermal sintering process helps to obtain biomaterials either in compact or in porous forms at relatively low temperatures in comparison to other sintering techniques.

By considering the influence of the processing parameters on the pore morphology and mechanical properties, high-pressure processing methods through hydrothermal sintering and freeze isostatic pressure techniques offer a new processing route for fabricating porous biomaterials. We have demonstrated the possibility of obtaining the porous biomaterials of amorphous SiO_2_ and Cu spheres by high-pressure processes. From our point of view, future work in this field should aim at tuning the processing parameters to obtain the desired microstructure and open interconnected porous biomaterials. By addressing the aforementioned points, it is possible to obtain porous biomaterials at high pressure with increased mechanical strength at low temperatures. Processing at low temperatures and high pressure will be an attractive option for materials that have OH components in their structure and thermosensitive materials. Furthermore, this will help to incorporate therapeutic molecules or other active molecules for targeted drug delivery. FIP and hydrothermal sintering for porous biomaterials is relatively new, and further detailed investigations on these innovative pressure processing methods will be very helpful in producing low-temperature porous biomaterials with increased mechanical strength.

In summary, the action of a solvent under pressure (hydrothermal) induces consolidation by dissolution-precipitation, even at minus temperatures, in order to initiate the intergranular cohesion or by using a template (ex H_2_O in FIP or PLA in HyS process). These high-pressure processes such as CIP in the presence of solvent, FIP and HyS could be applied to the manufacture of a biocomposite containing thermosensitive materials such as biopolymer (e.g., PLA) or pharmaceutical molecules.

## Figures and Tables

**Table 1:** High-pressure processing techniques used for materials processing.

	High Pressure Processes	Acronyms	Equipment (Tool)	Applications Material Processing	T (°C)	P (MPa)
**Vessel (Force isostatic)**	High Hydrostatic Pressing High Pressure Processing	HHP HPP	Tank, autoclave	Pascalization, decontamination, sterilization, disinfection of biological materials (Foods, Pharmacology, Medical)	20	xxx
	Cold isostatic Pressing	CIP	Tank, vessel	Compaction of powder	20	xxx
	Freeze Isostatic Pressing	FIP	Vessel	Consolidation of powder	T < 0 °C	xxx
	Autoclaving (Steam sterilization)		Autoclave, tank	Decontamination, sterilization, disinfection in medical	132	P < 1
	High Pressure (isostatic)	HyCG HyCr HyPu	Reactor, autoclave, bomb, vessel	Hydrothermal Crystal Growth Hydrothermal Crystallization Hydrothermal Purification	1000	xxx
	Hot Isostatic Pressing	HIP	Tank, autoclave, bomb	Compaction of powder, sintering	1000	xxx
	Reactive Hydrothermal Liquid-Phase Densification	rHLPD	Autoclave	Infiltration of permeable green compacts by aqueous solutions + reaction under hydrothermal conditions	240	unknown
	Hydrothermal Reaction-Sintering	HRS	Sealed capsule	Sintering of powder by hydrothermal oxidation of a metal + diffusion of H2 from the capsule + sintering of the oxide powder formed	900	xxx
**Piston –cylinder (Force on 1 axe)**	Uniaxial Pressing (ambient T)	UP	Non Leak-proof set-up: die, chamber, mold, cylinder, pelletizer	Compaction of powder	20	xxx
	Hydro Pressure Sintering (ambient T)	HyPS(≈HyS)	Leak-proof set-up	Compaction of powder, consolidation	20	xxx
	Cold Sintering Process(ambient T)	CSP (20 °C)	Non leak-proof set-up	Compaction of powder, consolidation	20	xxx
	High-pressure torsion	HPT	Anvils in rotation while pressing	Pre-compaction & subsequent consolidation	20	GPa
	Uniaxial pressing ultrasonic	PUA	Non Leak-proof set-up: mold	Compaction of powder by Uniaxial pressing + simultaneous powerful ultrasonic action	20	xxx
	Uniaxial Hot Pressing (dry materials)	UHP	Chamber = Non leak-proof set-up *(Heating by Induction RF exists)*	Sintering of powder	1000	xx
	Uniaxial Hot Pressing (humid materials)	HyS CSP (T > 20 °C)	Autoclave (2 openings) = Leak-proof set-up (*Heating by Induction RF exists*) Non leak-proof set-up	Sintering of powder Sintering of powder	500 200	xxx
	Hydrothermal Hot Pressing	HHP (=HyS)	Autoclave (2 openings) = Leak-proof set-up	Sintering of powder	250	xxx
	Oscillatory pressure sintering	OPS	Non leak-proof set-up: Graphite die	Sintering of powder	1300	xx + x
	High Pressure	HP-HT, HP-SPS	Belt, Bridgman	Sintering of powder	1800	GPa
**Multi-anvils (Force on multi axes)**	Ultra-high pressure sintering	UHPS	Multianvils (1 stage: 3 axes, 2 stages: Kawai, Walker)	Sintering of powder	2200	GPa
